# Prevalence and outcome of patients with non-ST segment elevation myocardial infarction with occluded “culprit” artery – a systemic review and meta-analysis

**DOI:** 10.1186/s13054-018-1944-x

**Published:** 2018-02-09

**Authors:** Chi-Sheng Hung, Ying-Hsien Chen, Ching-Chang Huang, Mao-Shin Lin, Chih-Fan Yeh, Hung-Yuan Li, Hsien-Li Kao

**Affiliations:** Department of Internal Medicine, National Taiwan University Hospital, 7 Chung-Shan South Road, Taipei, Taiwan

**Keywords:** Non-ST segment elevation myocardial infarction, Coronary occlusion, Electrocardiography

## Abstract

**Background:**

The aim was to determine the prevalence and impact of an occluded “culprit” artery (OCA) in patients with non-ST segment elevation myocardial infarction (NSTEMI).

**Methods:**

We searched PubMed, EMBASE, and Web of Science, with no language restrictions, up to 1 Jul. 2016. Observational cohorts or clinical trials of adult NSTEMI were eligible for inclusion to determine the prevalence if the proportion of OCA on coronary angiography was reported. Studies were further eligible for inclusion to determine the outcome if the association between OCA and clinical endpoints was reported.

**Results:**

Among the 60,898 patients with NSTEMI enrolled in 25 studies, 17,212 were found to have OCA. The average proportion of OCA in NSTEMI was 34% (95% CI 30–37%). Patients with OCA were more likely to have left circumflex artery as their culprit artery (odds ratio (OR) 1.65, 95% CI 1.15–2.37, *p* = 0.007), and this was associated with lower left ventricular ejection fraction (standard mean difference -0.29, 95% CI -0.34 to -0.34, *p* < 0.001), higher peak enzyme level (standard mean difference 0.43, 95% CI 0.27–0.58, *p* < 0.001), and higher risk for cardiogenic shock (OR 1.66, 95% CI 1.35–2.04, *p* < 0.001), compared with patients with a non-occlusive culprit artery. Death rate (OR 1.72, 95% CI 1.49–1.98, *p* < 0.001) and recurrent myocardial infarction (OR 1.7, 95% CI 1.06–2.75, *p* = 0.029) were also higher in patients with OCA, compared with patients with a non-occlusive culprit artery.

**Conclusions:**

Patients with OCA comprised a substantial portion of the NSTEMI population. These patients present with more severe symptoms and worse clinical outcome. Whether these patients should be treated with more aggressive strategy warrants further study.

**Electronic supplementary material:**

The online version of this article (10.1186/s13054-018-1944-x) contains supplementary material, which is available to authorized users.

## Background

Acute coronary syndrome (ACS) has been categorized into ST segment elevation myocardial infarction (STEMI) and non-ST segment elevation ACS (NSTEACS) based on the results of initial 12-lead electrocardiography (ECG). In patients with STEMI, early reperfusion therapy of the culprit artery is a class I indication in current guidelines [1]. In contrast, NSTEACS represent a wide spectrum of clinical syndromes, ranging from unstable angina (without cardiomyocyte loss) to NSTEMI (with cardiomyocyte necrosis). The management of NSTEACS is guided by risk stratification, with an early invasive strategy favoured in high-risk patients [2], especially for patients with positive cardiac necrosis biomarkers [3].

STEMI results from acute total or nearly total occlusion of a coronary artery [4]. However, totally occluded culprit artery (OCA) has also been observed among patients with NSTEACS. According to recent large retrospective studies, up to 30% of patients with NSTEACS had OCA [5, 6]. One of the reasons for OCA to present as NSTEACS, instead of STEMI, is that ECG is not sensitive enough to detect acute ischaemia or infarction over posterior or lateral walls, when the left circumflex artery (LCx) is usually the culprit artery. Indeed, the inferolateral territory was more frequently involved among patients with NSTEACS with OCA, compared with those with a non-occlusive culprit artery [6].

The clinical implication of OCA in NSTE-acute coronary syndrome (ACS) is still controversial, and how patients with NSTEACS with OCA should be treated is also unknown. Some studies report worse outcome for patients with OCA [5, 7–9], while other studies report otherwise [6, 10–12]. As unstable angina without positive cardiac markers was also included in some of these studies [6, 9], the observations on outcomes were inevitably variable. Given the high incidence of NSTEACS worldwide [13], and the potential impact of OCA on outcome, it is necessary to analyse in detail the currently available data. To decrease ambiguity, we limited our analysis to patients with positive cardiac necrosis biomarkers, i.e. NSTEMI. The goals of this meta-analysis were (1) to determine the proportion of patients with OCA in NSTEMI and (2) to determine the impact of OCA on the clinical severity and outcomes including death and recurrent myocardial infarction (MI) following NSTEMI.

## Methods

### Data sources and search strategies

This systemic review and meta-analysis was performed according to the preferred reporting items for systematic reviews and meta-analyses (PRISMA) guidelines [14]. A literature search was performed using PubMed, EMBASE, and Web of Science without restriction on language and year of publication. The following search terms were used in PubMed: (ACS or acute coronary syndrome[MeSH Terms] or (acute AND coronary AND syndrome) or “acute coronary syndrome” or NON-ST or NSTEMI or N-STEMI or NON-STEMI or NON STEMI or NSTEACS or angina, unstable[MeSH Terms]) AND ((“total occlusion” or “totally occluded” or (occluded AND culprit)) or “TIMI flow” or “TIMI-flow” or “(TIMI) flow”). Similar expressions were used in EMBASE and Web of Science.

### Eligibility criteria

#### Study design

Studies included in the analysis were prospective or retrospective cohorts or randomized controlled trials. The exclusion criteria were (1) abstracts, review articles, case-control studies and case series with the number of patients fewer than 20; (2) studies on myocardial infarction that only reported Q wave or non-Q wave MI; (3) studies on patients with unstable angina, stable coronary artery disease (CAD), or STEMI; (4) studies selecting patients by angiography results (for example, limited to single-vessel disease, left main disease, or chronic total occlusion); (5) studies that did not specify the result of cardiac necrosis biomarkers; (6) animal studies; and (7) duplicate reports.

#### Patients

The inclusion criteria were (1) adults aged 18 or above; (2) patients with chest pain; and (3) patients with elevated cardiac necrosis biomarkers (troponin or creatine kinase-MB isoenzyme (CK-MB)) according to the criteria specified in individual studies. Patients with STEMI or patients with NSTEACS and negative cardiac necrosis markers were excluded from analyses.

#### Proportion of totally occluded culprit artery

The determination of the culprit artery and the definition of OCA were based on the protocol of the individual study. Sensitivity analysis will be performed to include only studies defining OCA as Thrombolysis In Myocardial Infarction (TIMI)-flow grade 0 or 1 in the culprit artery (the most commonly used definition in these studies).

#### Outcome

Outcomes were assessed as all-cause mortality and recurrent MI. Endpoints were taken at 1 year or the longest follow up available (if the follow-up period was less than 1 year) in each study.

#### Data extraction

Extraction of data on study characteristics and outcomes was performed independently by two reviewers (CSH and YHC). Discrepancies between reviewers were resolved by consensus. The following data were extracted from each study enrolled in our analysis: the first author’s last name, year of publication, country of study performed, biomarkers measured, total number of patients, numbers of patients with OCA, mean age, gender, conventional risk factors for CAD (diabetes mellitus, hypertension, hyperlipidaemia, smoking) and outcome (recurrent MI or death). The study quality was judged using the Newcast-Ottawa scale (NOS) for observational studies [15]. This scale assesses the quality of the study based on patient selection, comparability and outcome. Studies controlled for age and sex were given one star for comparability; studies controlled for conventional risk factors for CAD were given another star for comparability. Studies with follow up of 1 year or longer were considered as long enough for outcome to occur. Studies with an NOS scale > =7 were considered of high quality.

#### Data analysis

We use the random-effects method because of its conservative summary estimate and because it incorporates between-study and within-study variance. We used the *I*^2^ statistic to assess heterogeneity of the event rates across studies [16]. A funnel plot with a linear regression approach to measure asymmetry was used to assess for the presence of publication bias [17]. Subgroup analyses were performed in different geographical regions (Asia-Pacific, European, North American, and multinational). Sensitivity analyses using the leave-one-out method were performed to identify key studies with major influence on between-study heterogeneity. Meta-regression was used to assess the influence of study characteristics on outcome. All statistical analyses were performed using Stata Version 13 software (Stata Corporation, Collage Station, TX, USA). All *p* values were two-sided, and those below 0.05 were regarded as statistically significant.

## Results

### Results of the search

**Fig. 1:**
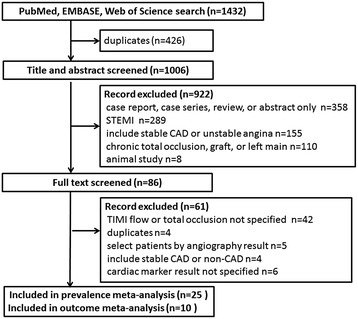
Searching strategy and number of studies at each stage of this meta-analysis. *STEMI* ST segment elevation myocardial infarction, *CAD* coronary artery disease, *TIMI* Thrombolysis In Myocardial Infarction

The initial search identified 1432 citations. After critical assessment of these papers, 25 studies fulfilled the inclusion criteria and were used to estimate the pooled proportion of OCA among NSTEMI patients. Ten of these 25 papers fulfilled the criteria and were used to analyse the impact of OCA on outcome (Fig. 1).

### Study characteristics

**Fig. 2:**
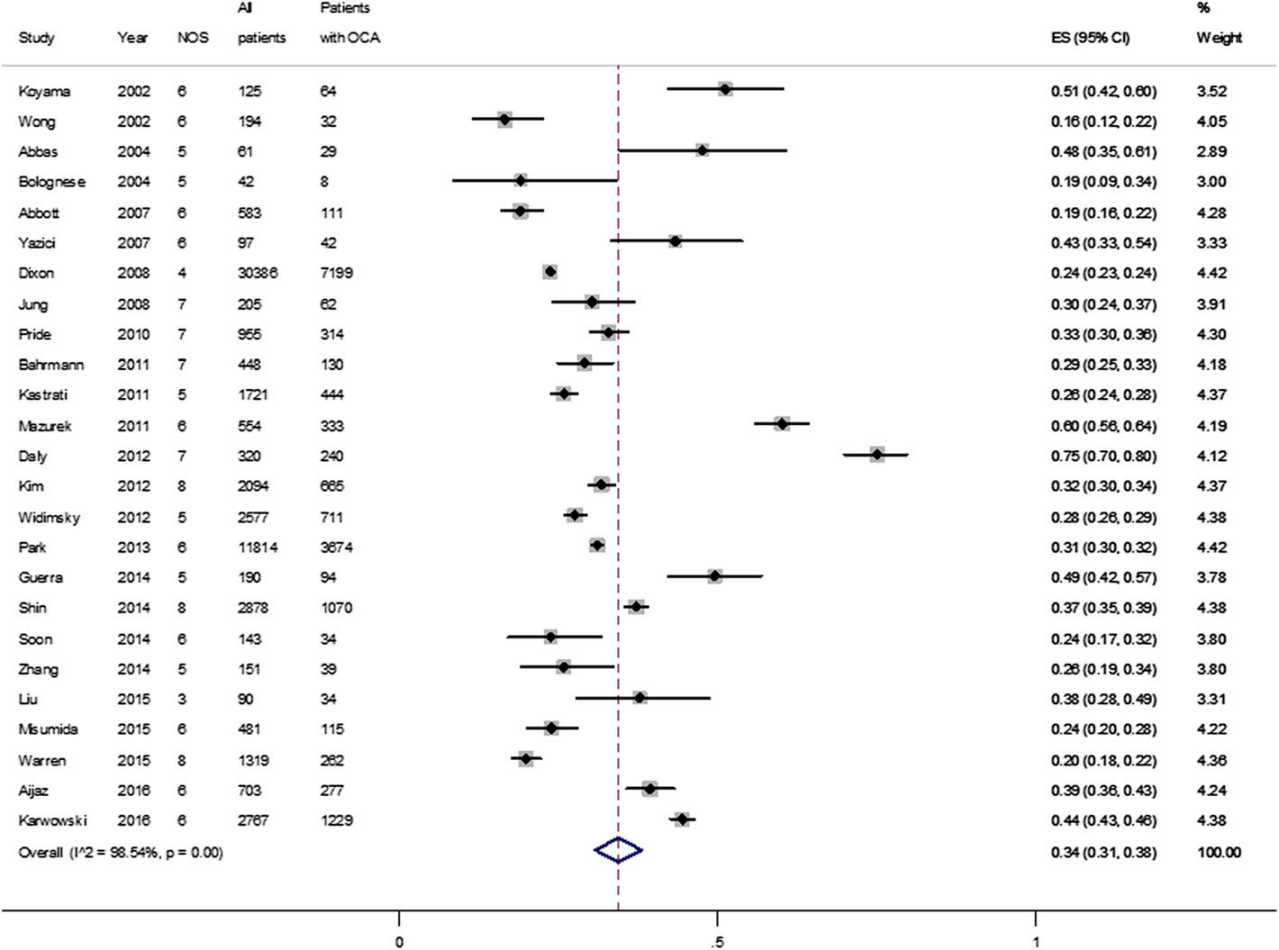
The pooled proportion of occluded culprit artery among patients with non-ST elevation myocardial infarction (number of studies = 25). *NOS* Newcastle-Ottawa scale, *OCA* occluded culprit artery, *ES* effect size

Characteristics of the 25 included studies are presented in Table 1. The studies were conducted in North American (*n* = 5), European (*n* = 8), Asia-Pacific (*n* = 10), or multinational regions (*n* = 2). The number of patients varied from 42 to 30,386, with mean age from 58 to 69 years, and 60 to 78.6% were male. Presence of hypertension was reported in 35 to 85.5% of patients, diabetes in 11.5 to 53.9%, hyperlipidaemia in 13 to 69.2%, and smoking in 21.3 to 80.3% of patients. Coronary artery bypass grafting (CABG) was excluded in five studies [5, 8, 18–20]. The definitions of OCA and exclusion criteria of the studies are summarised in Table 1. Among these 25 studies, 10 [5, 7–12, 21–23] reported distribution of the culprit artery, presence of cardiogenic shock, peak creatine kinase (CK) level, and outcome by the status of the culprit artery. These 10 studies were used for the analysis of the impact of OCA on presentation, death, and recurrent MI. The details of NOS for these 10 studies are presented in Table 2. The total scores of NOS for the other 15 studies (not included in outcome analysis) are presented in Fig. 2.

**Table 1:** Characteristics of 25 studies included in the pooled estimation of the proportion of occluded culprit arteries among patients with non-ST segment elevation myocardial infarction

References	Year	Area	Design^a^	Definition of OCA^b^	Number of patients	Age (years)	Male, *n* (%)	Multivessel disease, *n* (%)	OCA, *n* (%)	Major exclusion criteria	Follow-up duration	Outcome
Wong et al. [33]	2002	North America	1	1	194	62	138 (71.1%)	96 (49.5%)	32 (16.5%)	Increased risk of bleeding, LBBB		
Koyama et al. [34]	2002	Asia	2	1	125	61	93 (74.4%)	-	64 (51.2%)			
Bolognese et al. [35]	2004	Europe	2	1	42	63	33 (78.6%)	15 (35.7%)	8 (19%)	Cardiogenic shock		
Abbas et al. [36]	2004	North America	2	2	61	60	46 (75.4%)	28 (45.9%)	29 (47.5%)			
Yazici et al. [18]	2007	Asia	2	1	97	60	67 (69.1%)	-	42 (43.3%)	Heart failure, renal failure, prior CABG		
Abbott et al. [19]	2007	North America	2	1	583	62	363 (62.3%)	429 (73.6%)	111 (19%)	Prior CABG		
Jung et al. [10]	2008	Asia	3	1	205	63	123 (60%)	89 (43.4%)	62 (30.2%)	LBBB, Q wave formation	6 months	Death, MI
Dixon et al. [5]	2008	North America	2	1	30386	69	-	-	7199 (23.7%)	Prior CABG, prior MI	N/A	Death
Pride et al. [9]	2010	Multinational	1	1	955	63	646 (67.6%)	130 (13.6%)	314 (32.9%)	TIMI score <3, increased risk of bleeding	1 month	Death, MI
Bahrmann et al. [11]	2011	Europe	2	1	448	67	301 (67.2%)	276 (61.6%)	130 (29%)	Cardiogenic shock, increased risk of bleeding	6 months	Death, MI
Mazurek et al. [37]	2011	Europe	2	1	554	63	380 (68.6%)	-	333 (60.1%)			
Kastrati et al. [38]	2011	Europe	1	1	1721	68	661 (38.4%)	1377 (80%)	444 (25.8%)	Cardiogenic shock, malignancy, increased risk of bleeding		
Daly et al. [23]	2012	Europe	3	1	320	62	233 (72.8%)	38 (11.9%)	240 (75%)	LBBB	1 month	MACE
Kim et al. [7]	2012	Asia	2	2	2094	62	1467 (70.1%)	1298 (62%)	665 (31.8%)		1 year	Death
Widimsky et al. [39]	2012	Europe	3	1	2577	67	1735 (67.3%)	1708 (66.3%)	711 (27.6%)			
Park et al. [40]	2013	Asia	2	2	11814	65	7856 (66.5%)	(0%)	3674 (31.1%)			
Soon et al. [21]	2014	Pacific	3	4	143	64	97 (67.8%)	79 (55.2%)	34 (23.8%)	LBBB	1 year	Death, MI
Shin et al. [8]	2014	Asia	3	1	2878	64	1953 (67.9%)	1644 (57.1%)	1070 (37.2%)	LBBB, prior CABG	1 year	Death, MI
Zhang et al. [41]	2014	Asia	1	1	151	58	109 (72.2%)	-	39 (25.8%)	Increased risk of bleeding		
Guerra et al. [42]	2014	Europe	4	1	190	65	139 (73.2%)	-	94 (49.5%)	Crdiogenic shock, pericarditis, pacemaker		
Warren et al. [12]	2015	Multinational	1	1	1319	60	876 (66.4%)	-	262 (19.9%)	Cardiogenic shock, increased risk of bleeding	1 year	Death, MI
Liu et al. [43]	2015	Asia	2	1	90	-	-	-	34 (37.8%)			
Misumida et al. [44]	2015	North America	3	1	481	66	298 (62%)	253 (52.6%)	115 (23.9%)			
Aijaz et al. [22]	2016	Asia	4	1	703	58	565 (80.4%)	420 (59.7%)	277 (39.4%)		In-hospital stay	Death, MI
Karwowski et al. [20]	2016	Europe	2	1	2767	64	1796 (64.9%)	1330 (48.1%)	1229 (44.4%)	Prior MI, prior PCI, prior CABG		

*CABG* coronary artery bypass grafting, *LBBB* left bundle branch block, *MACE* major adverse cardiac events, *MI* myocardial infarction, *OCA* totally occluded culprit artery, *PCI* percutaneous coronary intervention, *N/A* not analysed

^a^Design: 1, randomized controlled trial; 2, prospective cohort; 3, retrospective cohort; 4, cross-sectional

^b^Definition of OCA: 1, Thrombolysis In Myocardial Infarction (TIMI) flow 0 or 1; 2, TIMI 0 only; 3, TIMI 1 only; 4,TIMI 0, TIMI 1, and TIMI 2

**Table 2:** Newcastle-Ottawa Scale (NOS) for assessing quality of observational studies

	Selection				Comparability		Outcome			Total
References	Representativeness of the exposed cohort	Selection of the non-exposed cohort	Ascertainment of exposure	Demonstration that outcome of interest was not present at start of study	Control for age and sex	Control for conventional CAD risk factors	Assessment of outcome	Was follow up long enough for outcomes to occur	Adequacy of follow up of cohorts	NOS quality scale
Jung et al. 2008 [10]	★	★	★	★	★	★	★			7
Dixon et al. 2008 [5]	★	★		★			★			4
Pride et al. 2010 [9]	★	★	★	★	★	★	★			7
Bahrmann et al. 2011 [11]	★	★	★	★	★	★	★			7
Daly et al. 2012 [23]	★	★	★	★	★	★	★			7
Kim et al. 2012 [7]	★	★	★	★	★	★	★	★		8
Soon et al. 2014 [21]	★	★	★	★			★	★		6
Shin et al. 2014 [8]	★	★	★	★	★	★	★	★		8
Warren et al. 2015 [12]	★	★	★	★	★	★	★	★		8
Aijaz et al. 2016 [22]		★	★	★	★	★	★			6

*CAD* coronary artery disease

### Prevalence of OCA in NSTEMI

All 25 studies provided data to be used for analysis of the proportion of OCA in patients with NSTEMI. A total of 17,212 patients had OCA among the 60,898 patients included. The average proportion of OCA in NSTEMI was therefore 0.34 (95% confidence interval (CI) 0.30–0.38) (Fig. 2). However, there was evidence of substantial heterogeneity in the proportion rate (*X*^2^ = 1644.9, degrees of freedom (df) = 24, *p* < 0.001, *I*^2^ = 98.54%). After excluding studies with OCA definition other than TIMI flow of 0 or 1, the average proportion of OCA in the remaining 20 studies was 0.35 (95% CI 0.30–0.41; *X*^2^ = 1067.58, df = 19, *I*^2^ = 98.22%, *p* < 0.001) (Additional file 1: Figure S1). After further excluding studies with time to diagnostic angiography more than 1 week or unknown, the average proportion of OCA in the remaining 14 studies was 0.35 (95% CI 0.28–0.41; *X*^2^ = 797.04, df = 13, *p* < 0.001, *I*^2^ = 98.37%) (Additional file 1: Figure S2).

The median proportion of patients with multivessel disease in all 25 studies was 57.6% (interquartile range 45.9–62.1%). Two studies reported a substantially lower proportion of patients with multivessel disease compared with other studies (13.6% in Pride 2010 [9] and 11.7% in Daly 2012 [23]). After excluding these two studies with lower proportions of patients with multivessel disease, the average proportion of OCA in the remaining 23 studies was 0.33 (95% CI 0.29–0.36; *X*^2^ = 1245.98, df = 22, *p* < 0.001, *I*^2^ = 98.23%) (Additional file 1: Figure S3).

Analysis by different locations showed possible heterogeneity among studies (Additional file 1: Figure S4). The average proportion of OCA was 0.42 (0.31–0.52) in Europe, 0.21 (0.18–0.24) in North America, 0.34 (0.31–0.38) in the Asia-Pacific area, and 0.24 (0.23–0.26) in multinational studies. We further divided the 25 studies into low (NOS = <5), moderate (NOS = 6), and high NOS (NOS > =7) studies. The average proportion of OCA was 0.30 (0.27–0.34, *I*^2^ = 92.3%) in low NOS studies, 0.35 (0.28–0.42, *I*^2^ = 98.2%) in moderate NOS studies, 0.36 (0.27–0.46, *I*^2^ = 98.7%) in high NOS studies. These results showed that subgroup analysis by different NOS did not reduce the heterogeneity (Additional file 1: Figure S5).

The leave-one-out sensitivity analyses could not identify studies with major impact on the between-study heterogeneity. The univariate meta-regression with the covariate of NOS, year of publication, age, proportion of patients with diabetes mellitus, hypertension, hyperlipidaemia and single-vessel disease, showed no significant impact on between-study heterogeneity (all *p* > 0.05).

### Severity and infarct size

Among the 10 studies used for analysis of outcome, severity scores (TIMI score or Global Registry of Acute Coronary Events (GRACE) score) were reported in 3 studies [7, 11, 12]. There was no difference in risk scores between patients with or without OCA. In five studies [5, 7, 10, 11, 21] that reported the occurrence of cardiogenic shock, the pooled odds ratio (OR) for cardiogenic shock was higher for patients with OCA (pooled OR 1.66, 95% CI 1.35–2.04, *p* < 0.001; *X*^2^ = 2.35, df = 4, *p* = 0.671) (Additional file 1: Figure S6). In six studies [7, 8, 10–12, 22] that reported the baseline left ventricular ejection fraction (LVEF) on admission, the standard mean difference in LVEF was -0.29 (95% CI -0.34 to -0.34, *p* < 0.001; *X*^2^ = 79.97, df = 5, *p* < 0.001) (Additional file 1: Figure S7) in patients with OCA, compared with patients with a non-occlusive culprit artery. In three studies [10, 11, 21] that reported peak CK level, the standard mean difference in CK was 0.43 (95% CI 0.27–0.58, *p* < 0.001; *X*^2^ = 1.36, df = 2, *p* = 0.506) in patients with OCA, compared with patients with a non-occlusive culprit artery (Additional file 1: Figure S8).

### Angiographic characteristics

Among the 10 studies used for analysis of outcome, the distribution of the culprit artery was reported in 6 studies [7, 8, 10–12, 21]. The pooled OR for LCx as the culprit artery was 1.65 (95% CI 1.15–2.37, *p* = 0.007; *X*^2^ = 38.5, df = 5, *p* < 0.001) (Additional file 1: Figure S9) in patients with OCA, compared with patients with a non-occlusive culprit artery. The distribution of infarct location was reported in 7 studies [5, 7, 8, 10–12, 21] among the 10 studied used for analysis of outcome. The pooled OR for infarct location in the posterior or lateral area was 2.24 (95% CI 1.63–3.09, *p* < 0.001; *X*^2^ = 75.45, df = 6, *p* < 0.001) for patients with OCA, compared with patients with a non-occlusive culprit artery (Additional file 1: Figure S10). The presence of collaterals on angiography had been reported in only one study [11], and well-developed collaterals were significantly more frequent among patients with OCA.

Among the 10 studies used for analysis of outcome, the information about revascularization was reported in 6 studies [5, 7, 8, 11, 21, 22] (Additional file 1: Table S1). In the three studies that reported the rate of successful percutaneous coronary intervention (PCI), the pooled OR for PCI success was 0.63 (95% CI 0.44–0.90, *p* = 0.011; X^2^ = 3.27, df = 2, *p* = 0.195) (Additional file 1: Figure S11) for patients with OCA, compared with patients with a non-occlusive culprit artery. In the three studies that reported stent length, the standard mean difference in stent length was 0.16 mm (95% CI 0.10–0.21, *p* < 0.001; *X*^2^ = 9.63, df = 2, *p* = 0.008) (Additional file 1: Figure S12) longer in patients with OCA, compared with patients with a non-occlusive culprit artery. In the three studies that reported stent length and the use of a drug-eluting stent, there was no significant difference between patients with OCA and patients with a non-occlusive culprit artery (Additional file 1: Figure S13 and S14).

### Predictors for NSTEMI with OCA

Among the 10 studies used for analysis of outcome, 5 [7, 9–11, 23] reported the predictors of the presence of OCA among patients with NSTEMI. Possible predictors included number of ST depression leads on 12-lead ECG [10], total ST depression score on 12-lead ECG [23], 80-lead body surface potential mapping [23], ECG abnormalities on inferolateral leads [7], peak CK-MB concentration [9, 10], fibrinogen at admission [10], dyslipidaemia [7], duration of continuous chest pain [10], and collateral supply [11]. The prediction of NSTEMI with OCA had only been evaluated by total ST depression score on 12-lead ECG (*c* statistic 0.693; 95% CI 0.521–0.771, *p* = 0.058) and 80-lead body surface potential mapping (*c* statistic 0.906; 95% CI 0.838–0.983, *p* < 0.001) among these possible predictors [23].

Risk stratification scores had been reported in four studies, with TIMI score reported in three [7, 10, 21] and GRACE score reported in one study [11]. There was no significant difference between patients with and without OCA using these two risk scores.

### Outcome

**Fig. 3:**
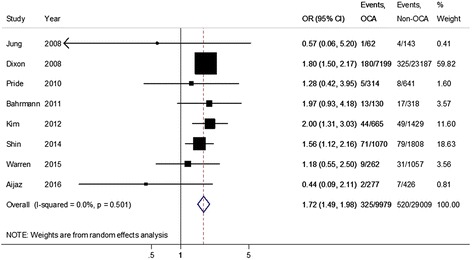
Pooled odds ratio for all-cause mortality among patients with non-ST segment elevation myocardial infarction and an occluded culprit artery (OCA) compared to with those with a non-occluded culprit artery (Non-OCA)

Among the 10 studies used for analysis of outcome, mortality was reported in 8 studies [5, 7–12, 22]. The pooled OR for mortality was 1.72 (95% CI 1.49–1.98, *p <* 0.001; *X*^2^ = 6.34, df = 7, *p* = 0.501) for patients with OCA compared with patients with a non-occlusive culprit artery (Fig. 3). After excluding studies without clear OCA definition, the pooled OR from the remaining six studies was 1.46 (95% CI 1.12–1.90, *p* = 0.006; *X*^2^ = 4.07, df = 5, *p* = 0.539) (Additional file 1: Figure S15). Further excluding studies with time to diagnostic angiography more than 28 days or unknown, the pooled OR from the remaining four studies was 1.52 (95% CI 1.15–2.01, *p* = 0.003; *X*^2^ = 1.67, df = 3, *p* = 0.644) (Additional file 1: Figure S16).

**Fig. 4:**
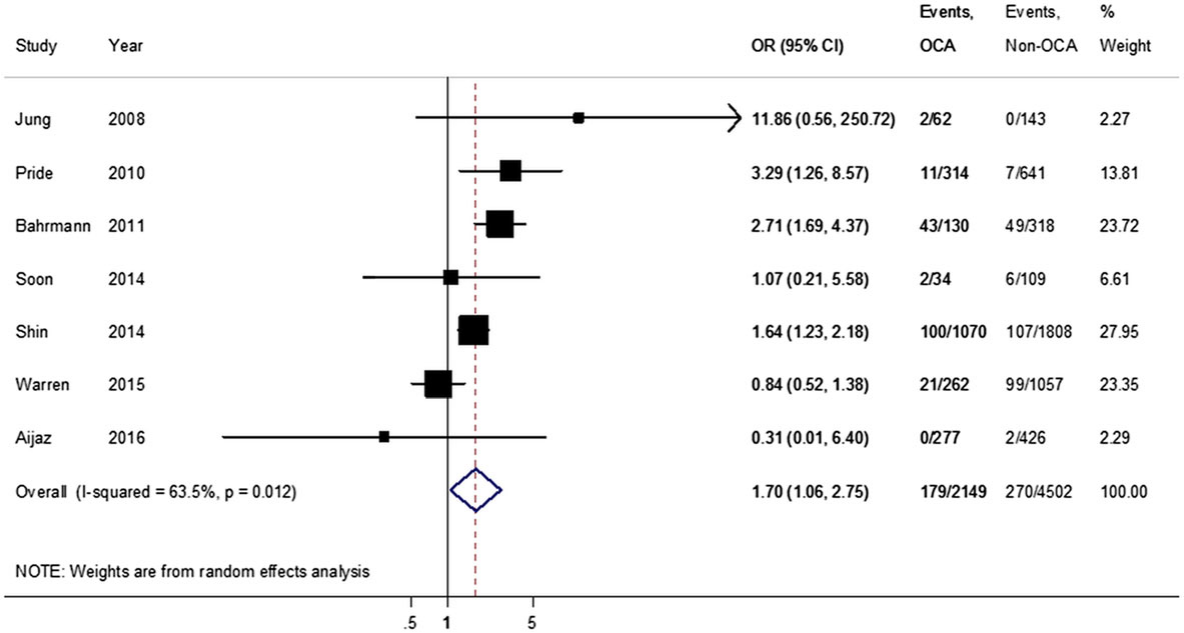
The pooled odds ratio for recurrent myocardial infarction among patients with non-ST segment elevation myocardial infarction and an occluded culprit artery (OCA) compared to with those with a non-occluded culprit artery (Non-OCA)

The recurrence of MI was reported in seven studies [8–12, 21, 22]. The pooled OR for recurrent MI was 1.7 (95% CI 1.06–2.75, *p* = 0.029; *X*^2^ = 16.43, df = 6, *p* = 0.012) for patients with OCA compared with patients with a non-occlusive culprit artery (Fig. 4). Further excluding studies without a clear OCA definition, the pooled OR from the remaining six studies was 1.77 (95% CI 1.06–2.95, *p* = 0.03; *X*^2^ = 16.16, df = 5, *p* = 0.006) (Additional file 1: Figure S17). Further excluding studies with time to diagnostic angiography more than 28 days or unknown, the pooled OR from the remaining four studies was 1.67 (95% CI 0.94–2.95, *p* = 0.079; *X*^2^ = 12.95, df = 3, *p* = 0.005) (Additional file 1: Figure S18).

### Test for publication bias

**Fig. 5:**
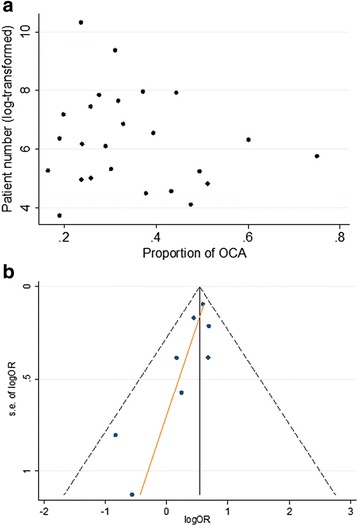
Funnel plot. (**a**) Pooled proportion. (**b**) Outcome. *OCA* occluded culprit artery, *OR* odds ratio

The funnel plots for studies reporting the proportion of OCA (by sample size vs. proportion) were asymmetrical (Fig. 5a). The Egger test confirmed the presence of small-study effects (*p* < 0.001). The small-study effect persisted even when excluding studies with a less strict OCA flow definition and studies with time to angiography more than 1 week (Egger test *p* < 0.001). The funnel plot for studies reporting on outcome suggested publication bias (Egger *p* < 0.001) (Fig. 5b).

## Discussion

This meta-analysis showed that (1) the overall estimated proportion of OCA in NSTEMI was 34%; (2) NSTEMI with OCA was associated with more LCx as the culprit artery, higher peak enzyme level, lower LVEF, and more cardiogenic shock; and (3) NSTEMI with OCA was associated with a higher rate of recurrent MI and death. These important findings remained the same if we limited our analysis to studies using TIMI flow 0–1 as the definition of OCA and timely diagnostic angiography after onset.

There is substantial heterogeneity in our analysis, with a wide range of proportion of OCA reported in the studies included (0.15–0.75). The explanations for this variation include differences in the study geographic region, study design and OCA definition, patient population, and timing of angiography. The standard initial ECG evaluation protocol is often unavailable in retrospective studies, and subtle ST change, especially in the posterior leads, may be missed. Some of the studies excluded patients with prior coronary artery bypass grafting (CABG) [5, 8, 18–20]. This difference in enrolment criteria may also influence the proportion of patients with OCA. The angiographic finding may change with time after symptom onset [24], but the estimated OCA proportion and outcome did not change after excluding studies with angiography performed more than 7 days after onset. Meta-regression did not identify the conventional risk factors as the source of the heterogeneity in the pooled estimation of OCA proportion. We did find different proportions among studies in different geographic regions. Ethnic difference has been reported in the prevalence of coronary artery disease [25, 26] and in the distribution of coronary lesions [27]. However, whether the difference between geographic regions reflected a real difference in the ethnicity or a combination of different design, inclusion criteria, and timing of angiography, is not clear.

Our analysis suggested that OCA represents a substantial portion of NSTEMI, and carries a worse clinical outcome. Some researchers proposed that this condition should be considered as “STEMI equivalent” and treated as such [6, 7]. However, whether OCA mandates a more aggressive treatment strategy or simply reflects worse disease severity cannot be determined from the current analysis. There are several possible causes of OCA in NSTEMI: (1) acute total occlusion of vessel suppling some part of myocardium (esp lateral wall) which does not consistently lead to ST elevation in conventional 12-lead ECG, probably due to the absence of corresponding leads; (2) acute total occlusion of vessels with good collaterals; (3) acute total occlusion in the territory with dual blood supply [28]; (4) acute total occlusion with a small infarction area; or (5) chronic total occlusion misclassified as acute occlusion. In the first condition, timely reperfusion by primary percutaneous coronary intervention may improve the outcome as in STEMI. Although our analysis cannot provide evidence for any of the aforementioned hypotheses, the higher percentage of LCx as the culprit artery suggests that acute total occlusion not detected by standard 12-lead ECG may be the most possible cause. Acute LCx occlusion can result in isolated posterior infarction with ST elevation only detected in leads V7–V9 [29]. The isolated posterior infarction due to LCx occlusion is not uncommonly missed by medical personnel [30], and is associated with longer time to reperfusion [31] and is less likely to be treated by primary percutaneous coronary intervention [32]. These factors are possibly associated with less favourable outcome, as in our analyses. Collateral artery status was described in only one study, reporting higher incidence of angiographic collaterals in NSTEMI with OCA [11]. The authors also identified better outcome in these patients with OCA with collaterals, compared with those without. Further study on the impact of collateral in patients with NSTEMI with OCA is needed. Finally, determination of the culprit artery is sometimes difficult in NSTEMI without diagnostic ECG. Hence, misclassifying a chronic total occlusion as the culprit is possible clinically, and the true incidence of misclassified chronic total occlusion cannot be determined by our analysis.

Considering the large number of patients with NSTEMI worldwide [13], it is imperative to explore methods to identify patients with NSTEMI with OCA. Three studies included in the present meta-analysis reported the TIMI or GRACE risk score, but these scores cannot differentiate the outcomes of patients with OCA or those with a non-occlusive culprit artery. Body surface potential mapping using 80 chest leads may improve detection of ST segment elevation [23], but its clinical practicality needs further investigation. The LVEF assessed by echocardiography was significantly lower in patients with NSTEMI with OCA than in patients with a non-occlusive culprit artery according to our analysis. Whether LVEF helps to identify patients with NSTEMI with OCA should be validated prospectively. Other practices incorporating coronary computed tomography angiography, echocardiography, or routine posterior-leads ECG may all be helpful, but again mandating future research to establish their clinical significance.

Our analysis has several strengths. First, we enrolled only patients with elevated cardiac necrosis markers. Focusing our biomarker-positive NSTEACS population helps us to decrease the ambiguity, targeting only patients with objective myocardial injury. More importantly, patients with biomarker-positive NSTEACS may benefit from an early invasive strategy, compared with biomarker-negative patients [3]. Second, the potential influence of OCA definition and angiography timing were well-considered and controlled. By conducting sensitivity analyses, we showed that there was no major impact of these two factors on the heterogeneity of OCA incidence.

Our analysis does have several limitations. First, the ECG protocol used was not reported in detail in most of the studies. Whether posterior leads have been applied in individual studies was unclear. Second, patients with contraindication for angiography or percutaneous coronary intervention were excluded by the analysis design. Therefore our results cannot be generalized in this population. Third, angiography in the included studies was done within one week after symptom onset. Hence the true OCA incidence at the time of onset could not be deducted from the present analysis. Fourth, a certain proportion of patients with posterior STEMI may be classified as having NSTEMI. This misclassification may be improved in the future by including routine posterior leads to detect posterior STEMI in patients with chest pain. Fifth, because the outcomes reported in the studies had not been stratified by some important moderators including the procedures and the number of diseased vessels, further analysis on the impact of OCA according to these moderators cannot be performed. Finally, given the asymmetry of funnel plot, we cannot exclude the potential publication bias against studies finding no significant difference in the outcomes of NSTEMI with or without OCA.

## Conclusion

Approximately one third of patients with NSTEMI have OCA. These patients present with more severe clinical symptoms and have a worse outcome, compared with those with a non-occlusive culprit artery. There is as yet no reliable tool to identify this group of patients before performing angiography. Whether timely reperfusion will benefit this group of patients warrants further studies.

## Additional file

Additional file 1: Supplementary figures and table. (DOCX 3836 kb)

## Abbreviations

ACS: Acute coronary syndrome
CABG: Coronary artery bypass grafting
CAD: Coronary artery disease
CK: Creatine kinase
CK-MB: Creatine kinase-MB isoenzyme
ECG: Electrocardiography
GRACE: Global Registry of Acute Coronary Events
LCx: Left circumflex artery
LVEF: Left ventricular ejection fraction
MI: Myocardial infarction
NOS: Newcastle–Ottawa scale
NSTEACS: Non-ST segment elevation acute coronary syndrome
NSTEMI: Non-ST segment elevation myocardial infarction
OCA: Occluded culprit artery
OR: Odds ratio
PCI: Percutaneous coronary intervention
STEMI: ST segment elevation myocardial infarction
TIMI: Thrombolysis In Myocardial Infarction
