# Supplementary material for: Prevalence and outcome of patients with non-ST segment elevation myocardial infarction with occluded “culprit” artery – a systemic review and meta-analysis
Source: Crit Care. 2018 Feb 9;22:34. doi: 10.1186/s13054-018-1944-x (PMC5806289; doi:10.1186/s13054-018-1944-x)
Supplement: Supplementary file 1 — Supplementary figures and table. (DOCX 3836 kb) [file 13054_2018_1944_MOESM1_ESM.docx]

**Supplemental materials**

# Figure S1: The pooled proportion of occluded culprit artery among patients with non-ST elevation myocardial infarction using the TIMI flow 0/1 as the definition of occluded culprit artery (number of study =20)


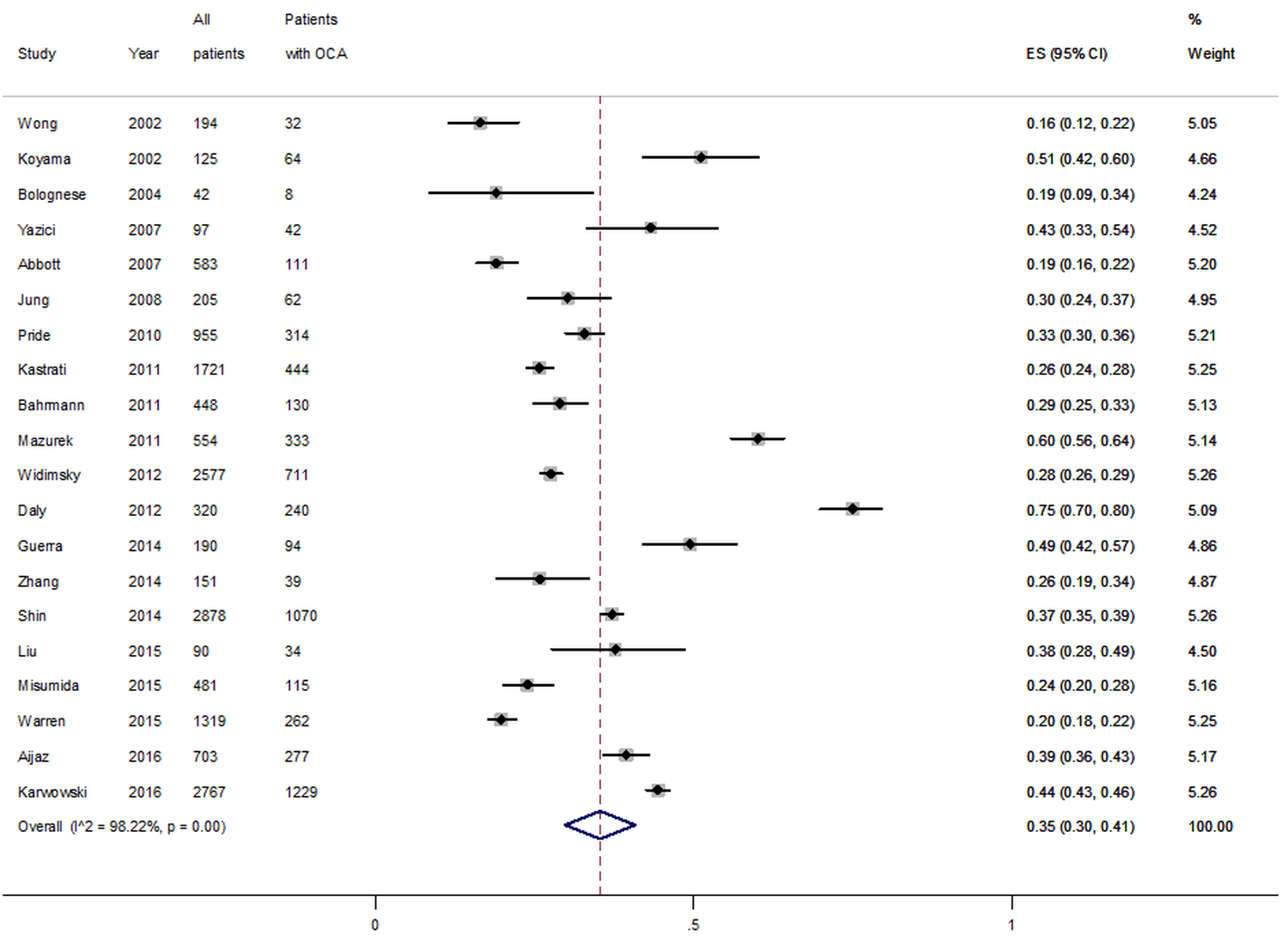


# Figure S2: The pooled proportion of occluded culprit artery among patients with non-ST elevation myocardial infarction using the TIMI flow 0/1 as the definition of occluded culprit artery and time to coronary angiography within 7 days (number of study =14)


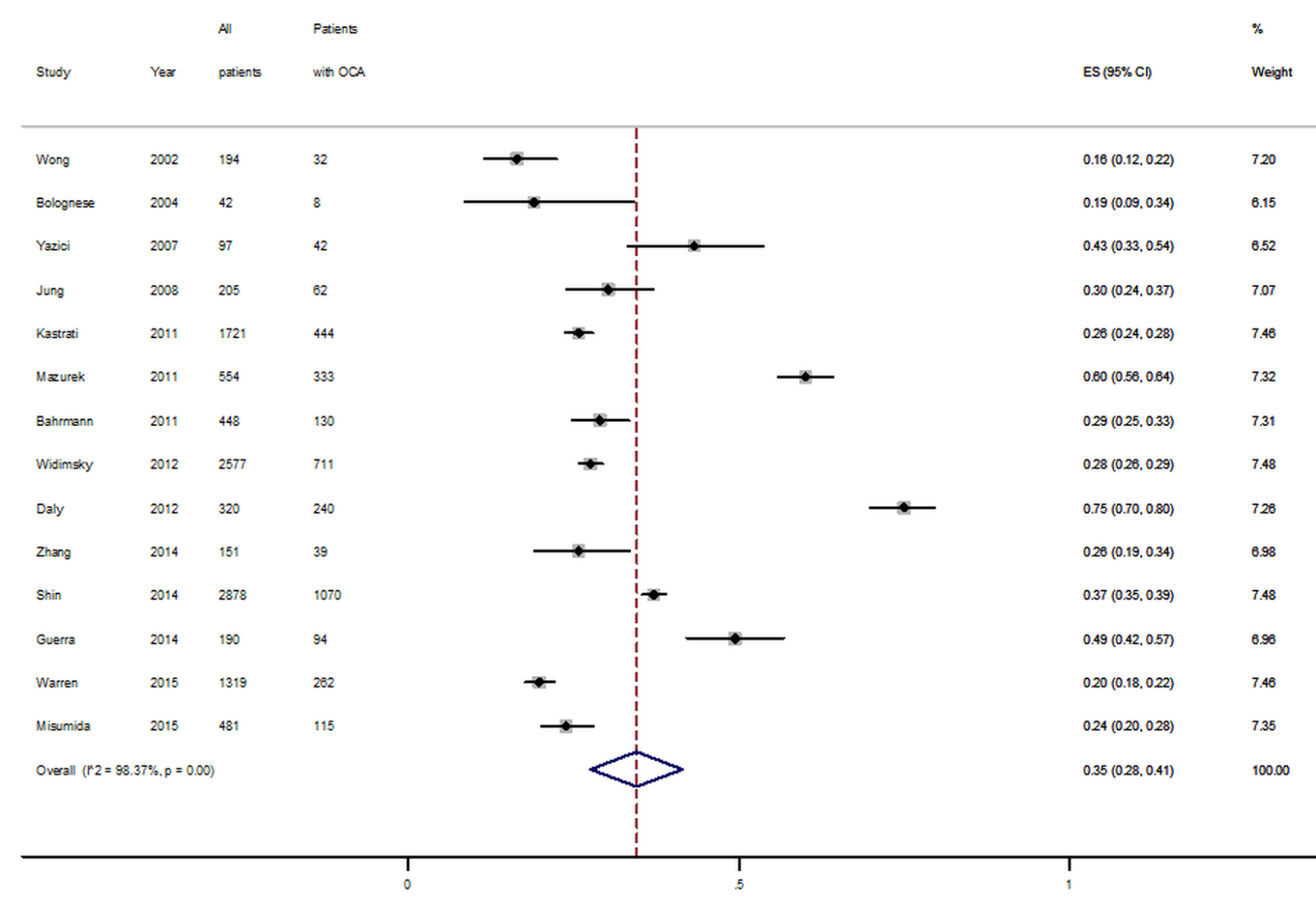


# Figure S3: The pooled proportion of occluded culprit artery among patients with non-ST elevation myocardial infarction after excluding two studies with substantially lower proportion of patients with multivessel disease.

# Figure S4: The pooled proportion of occluded culprit artery among patients with non-ST elevation myocardial infarction by different study locations.


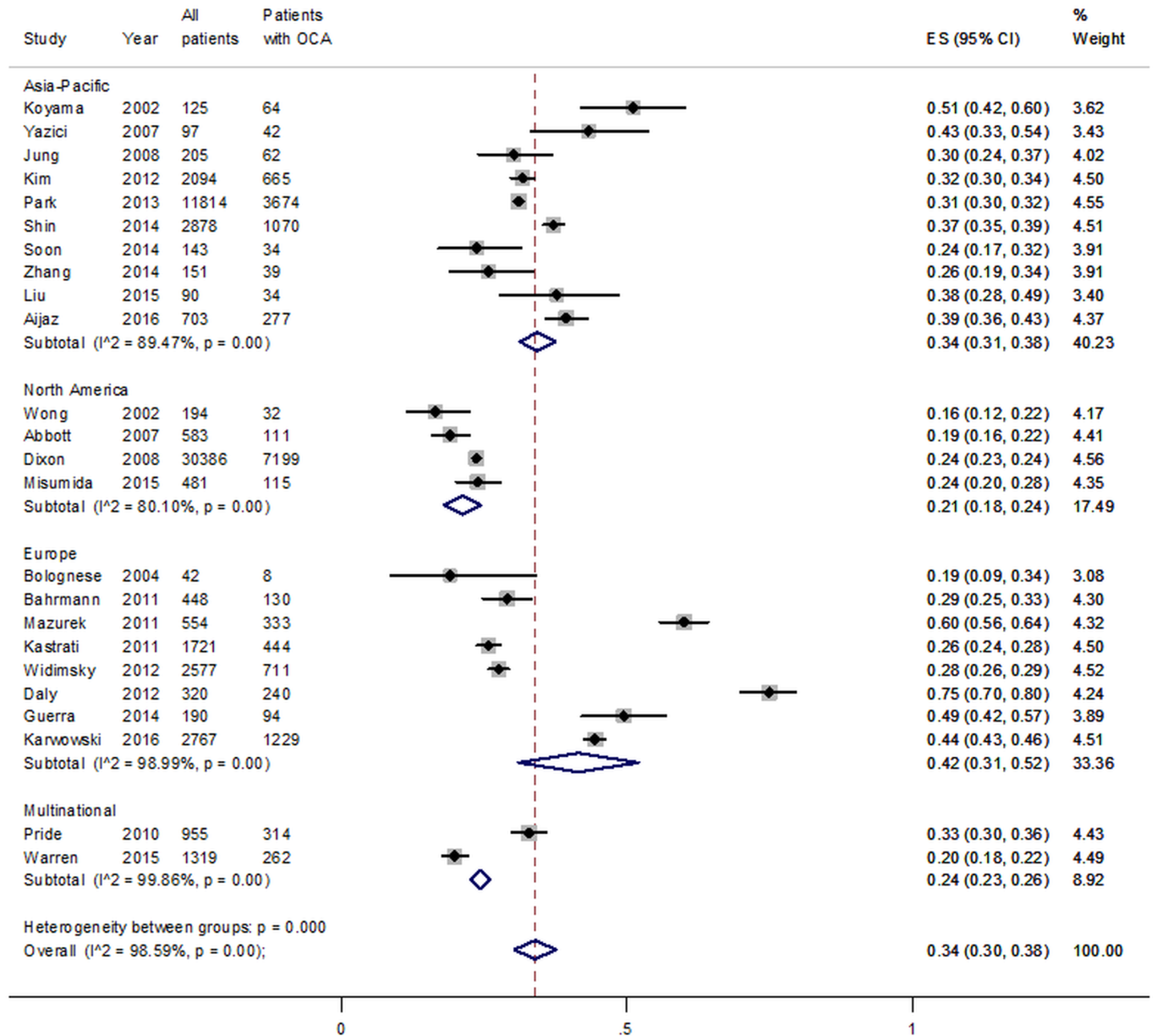


# Figure S5: The pooled proportion of occluded culprit artery among patients with non-ST elevation myocardial infarction by different Newcastle-Ottawa scale (NOS). Group 1: low NOS studies (NOS=<5); Group 2: moderate NOS studies (NOS=6); Group 3: high NOS studies (NOS>=7).


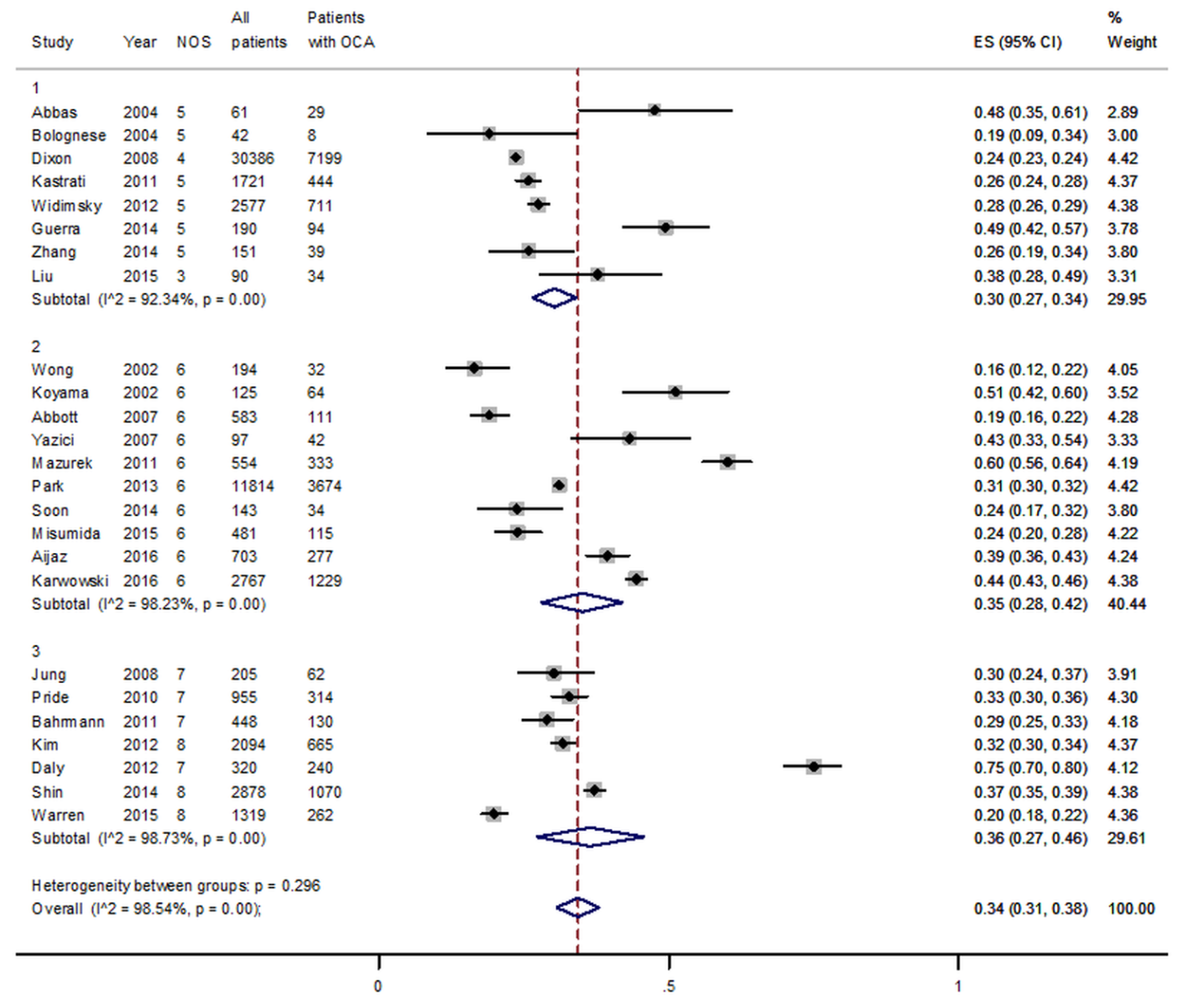


# Figure S6: The pooled odds ratio for cardiogenic shock among patients with non-ST elevation myocardial infarction and occluded culprit artery compared with those with non-occluded culprit artery


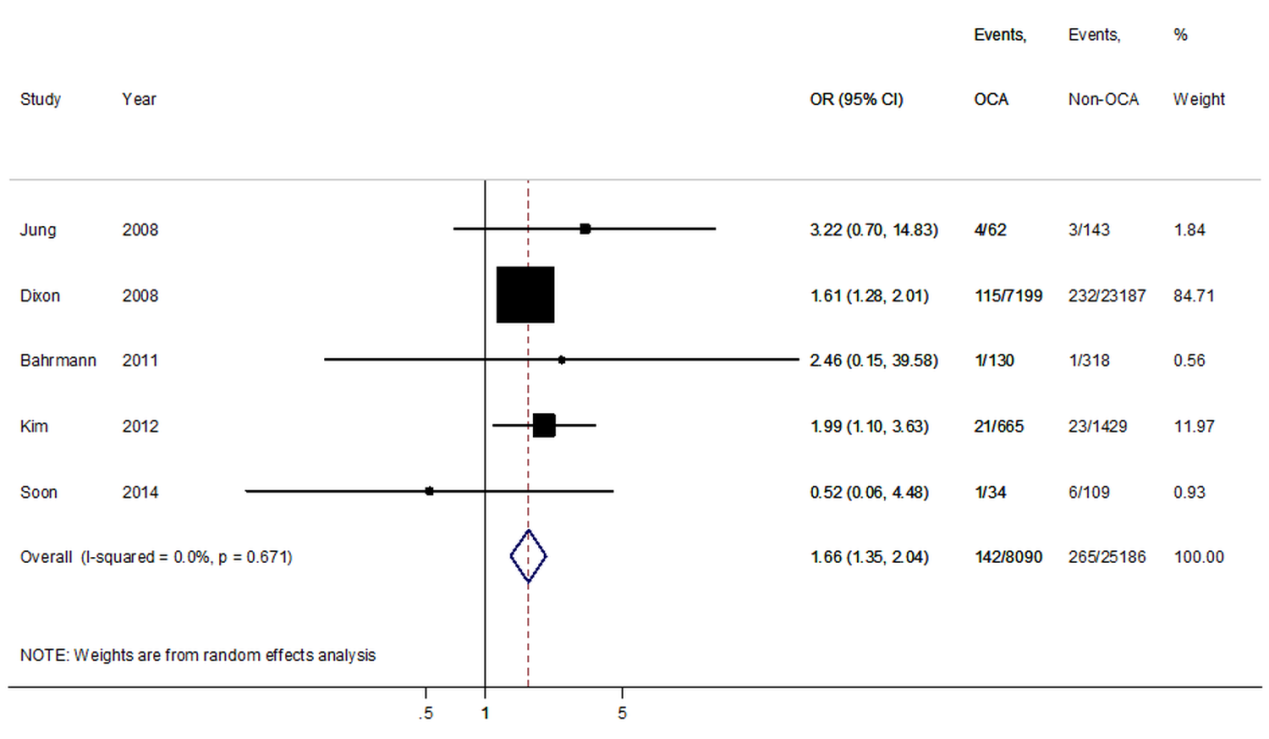


# Figure S7: The standardized mean difference for left ventricular ejection fraction among patients with non-ST elevation myocardial infarction and occluded culprit artery compared with those with non-occluded culprit artery


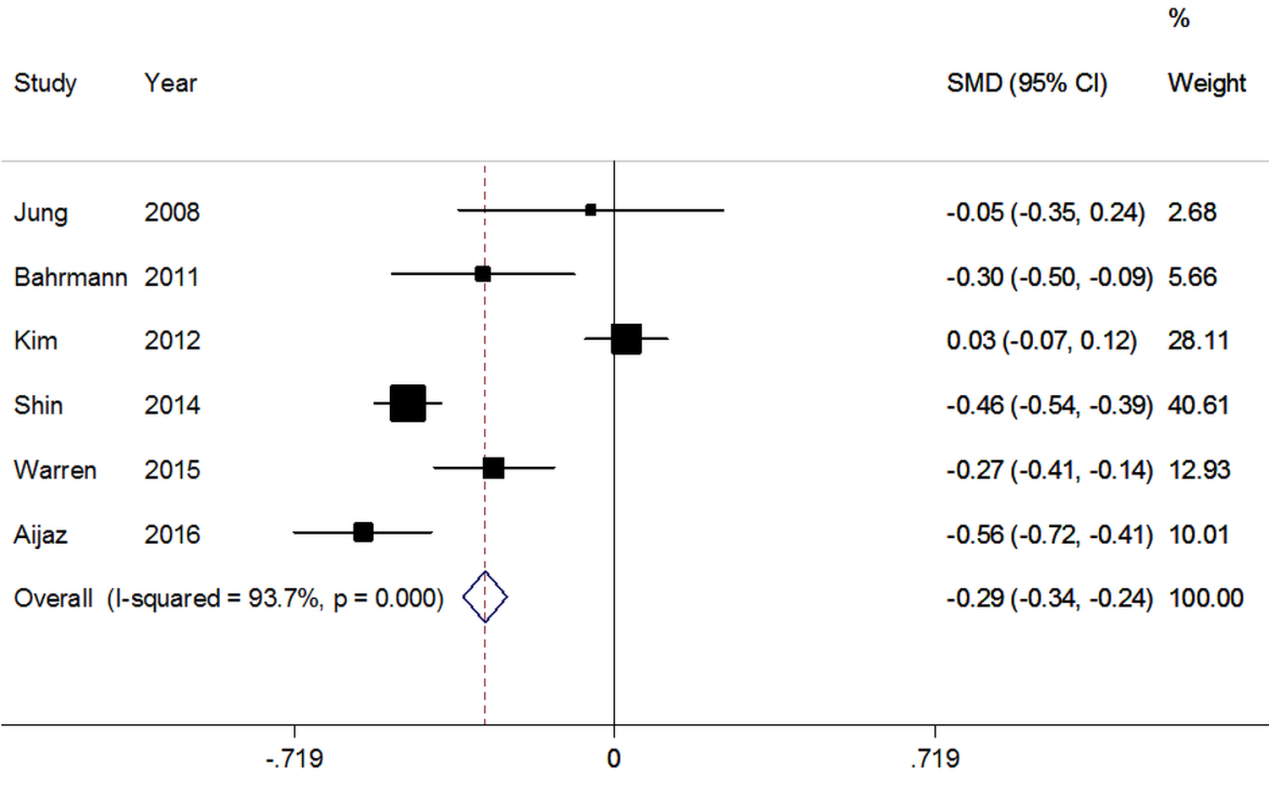


# Figure S8: The standardized mean difference for peak creatine kinase among patients with non-ST elevation myocardial infarction and occluded culprit artery compared with those with non-occluded culprit artery


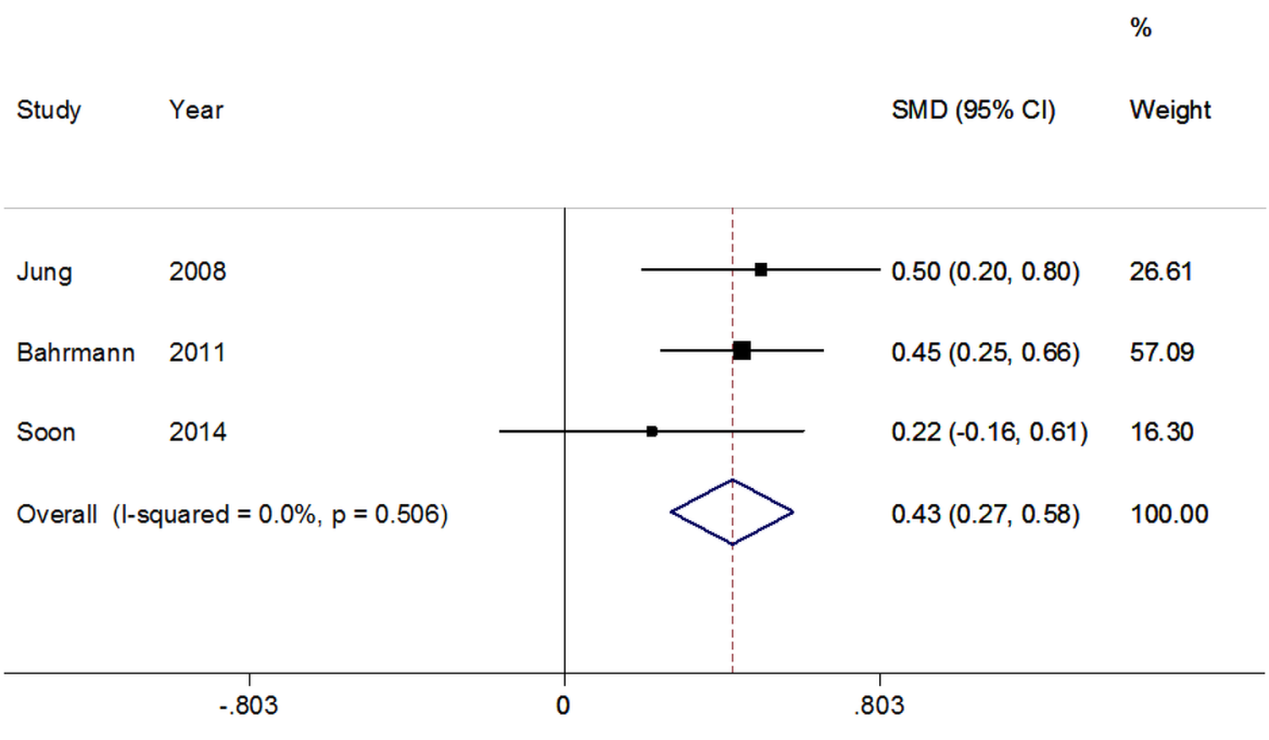


# Figure S9: The pooled odds ratio for left circumflex artery as the culprit artery among patients with non-ST elevation myocardial infarction and occluded culprit artery compared with those with non-occluded culprit artery


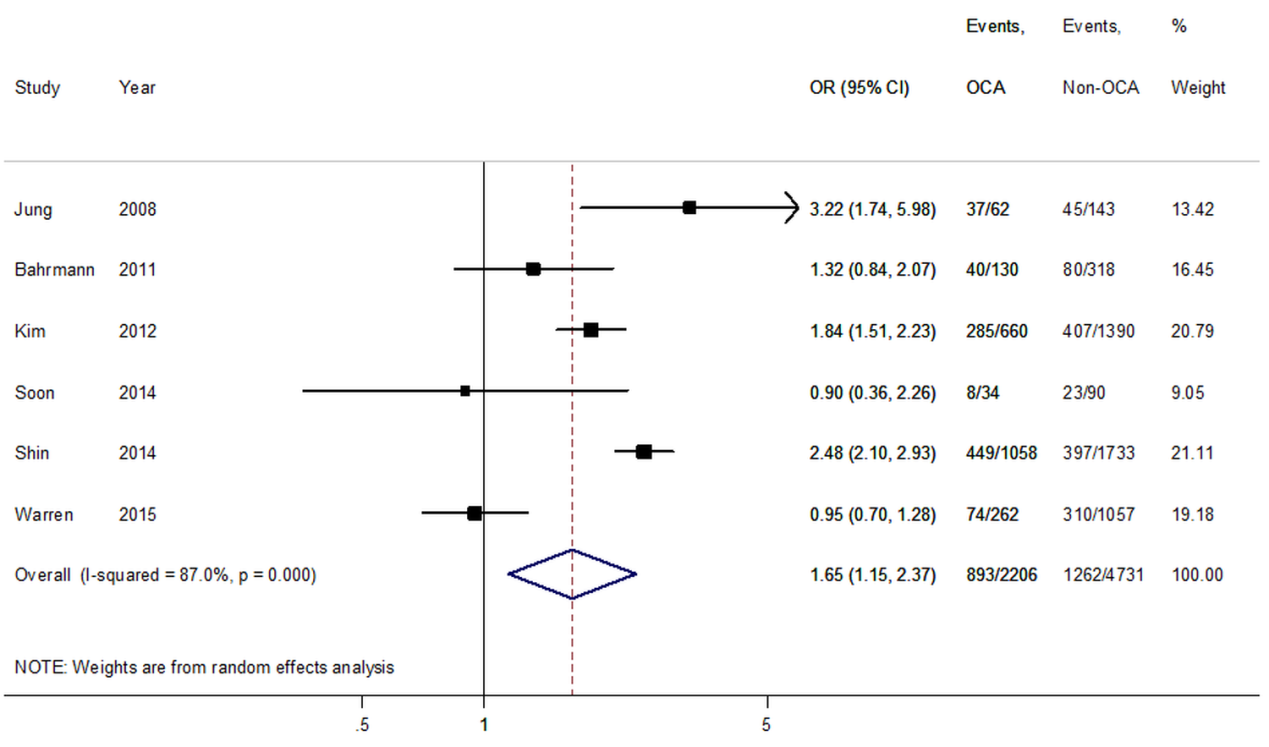


# Figure S10: The pooled odds ratio for inferolateral area as the infarction area among patients with non-ST elevation myocardial infarction and occluded culprit artery compared with those with non-occluded culprit artery


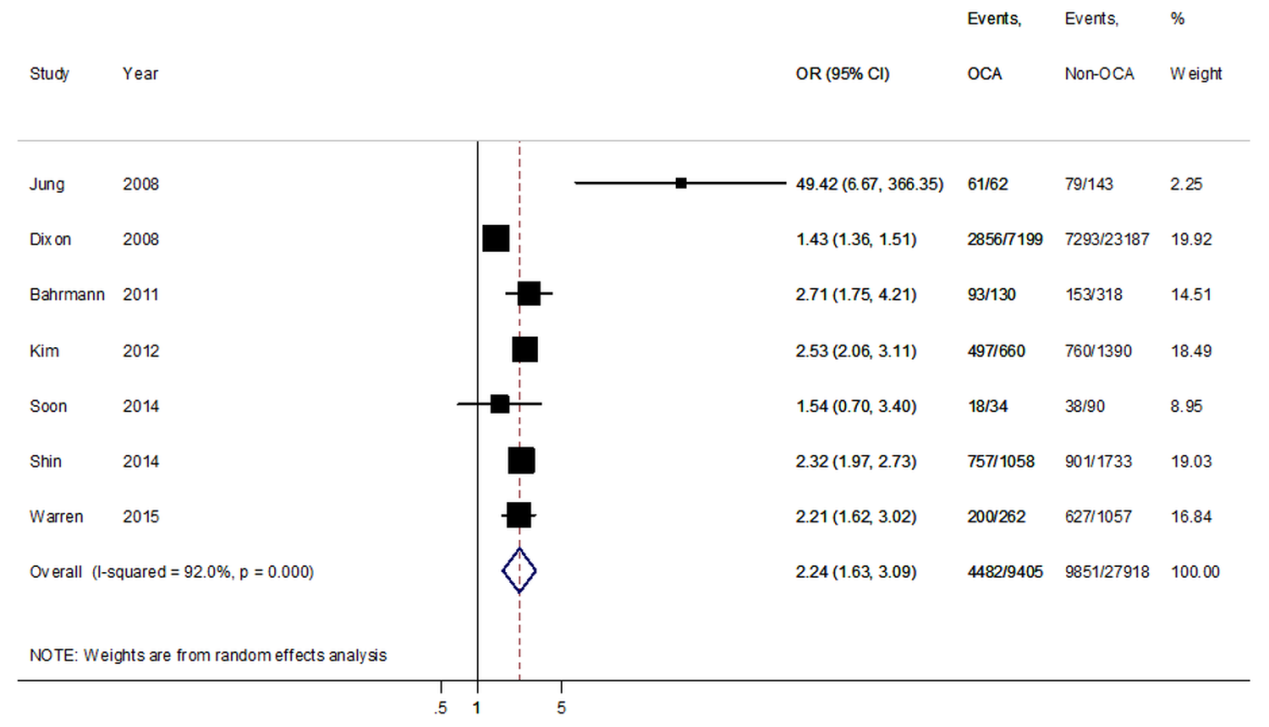


# Figure S11 The pooled odds ratio for successful percutaneous coronary intervention among patients with non-ST elevation myocardial infarction and occluded culprit artery compared with those with non-occluded culprit artery


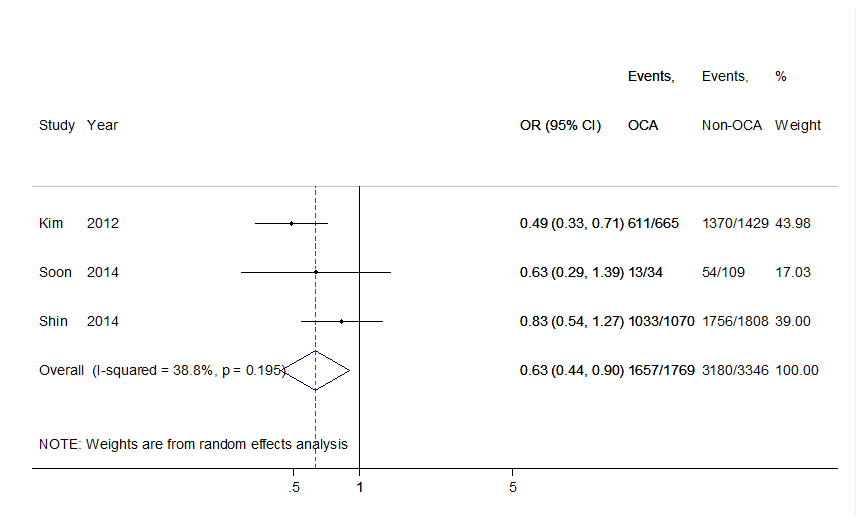


# Figure S12 The standardized mean difference for the total stent length among patients with non-ST elevation myocardial infarction and occluded culprit artery compared with those with non-occluded culprit artery


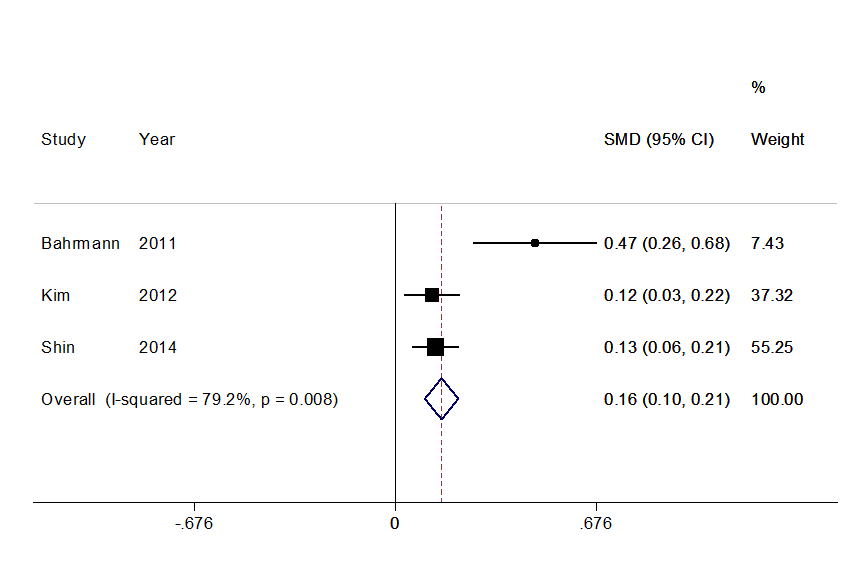


# Figure S13 The standardized mean difference for the number of stent used among patients with non-ST elevation myocardial infarction and occluded culprit artery compared with those with non-occluded culprit artery


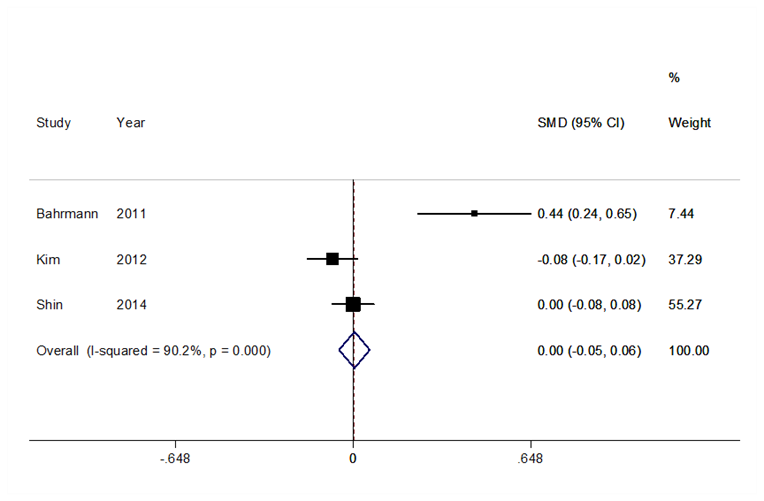


# Figure S14 The pooled odds ratio for the use of drug-eluting stent among patients with non-ST elevation myocardial infarction and occluded culprit artery compared with those with non-occluded culprit artery


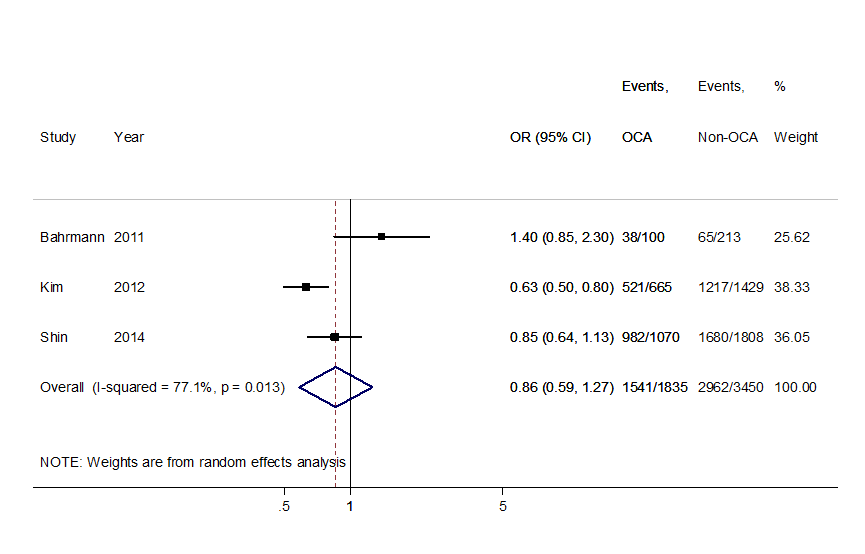


# Figure S15: The pooled odds ratio for all-cause mortality among patients with non-ST elevation myocardial infarction and occluded culprit artery compared with those with non-occluded culprit artery using the TIMI flow 0/1 as the definition of occluded culprit artery.


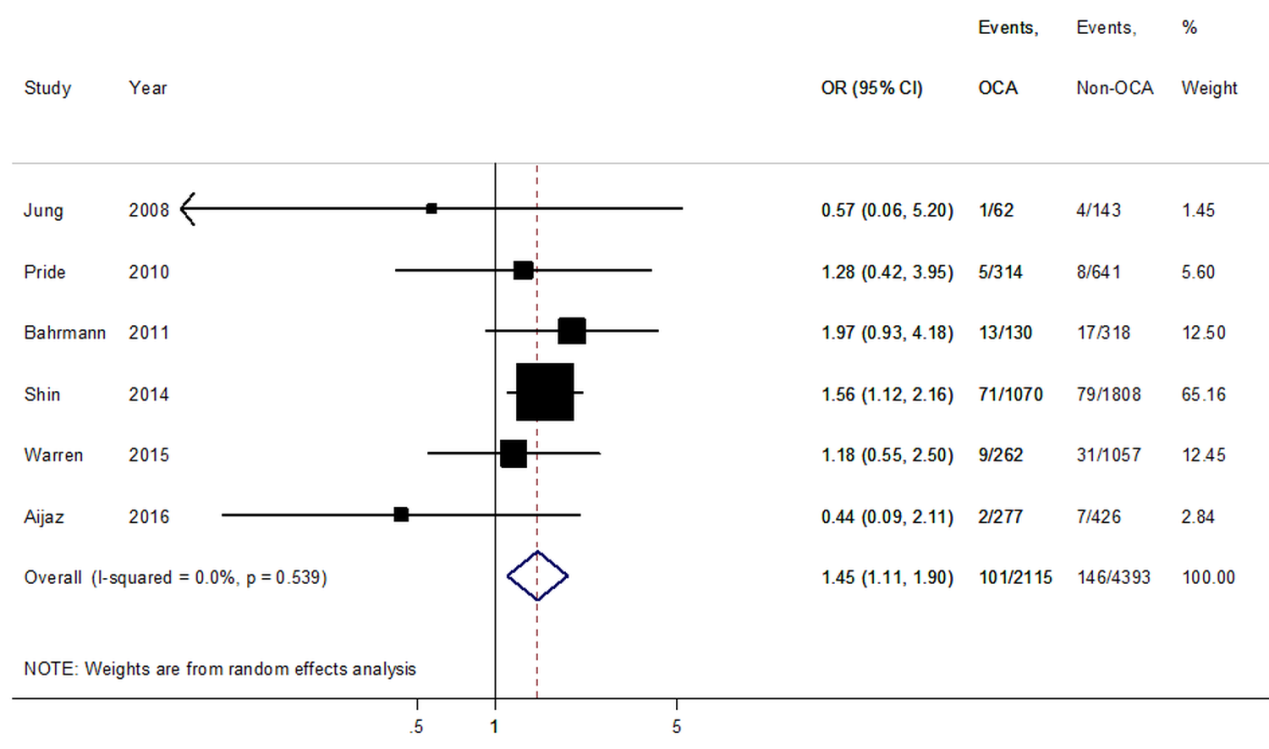


# Figure S16: The pooled odds ratio for all-cause mortality among patients with non-ST elevation myocardial infarction and occluded culprit artery compared with those with non-occluded culprit artery using the TIMI flow 0/1 as the definition of occluded culprit artery and time to coronary angiography within 28 days.


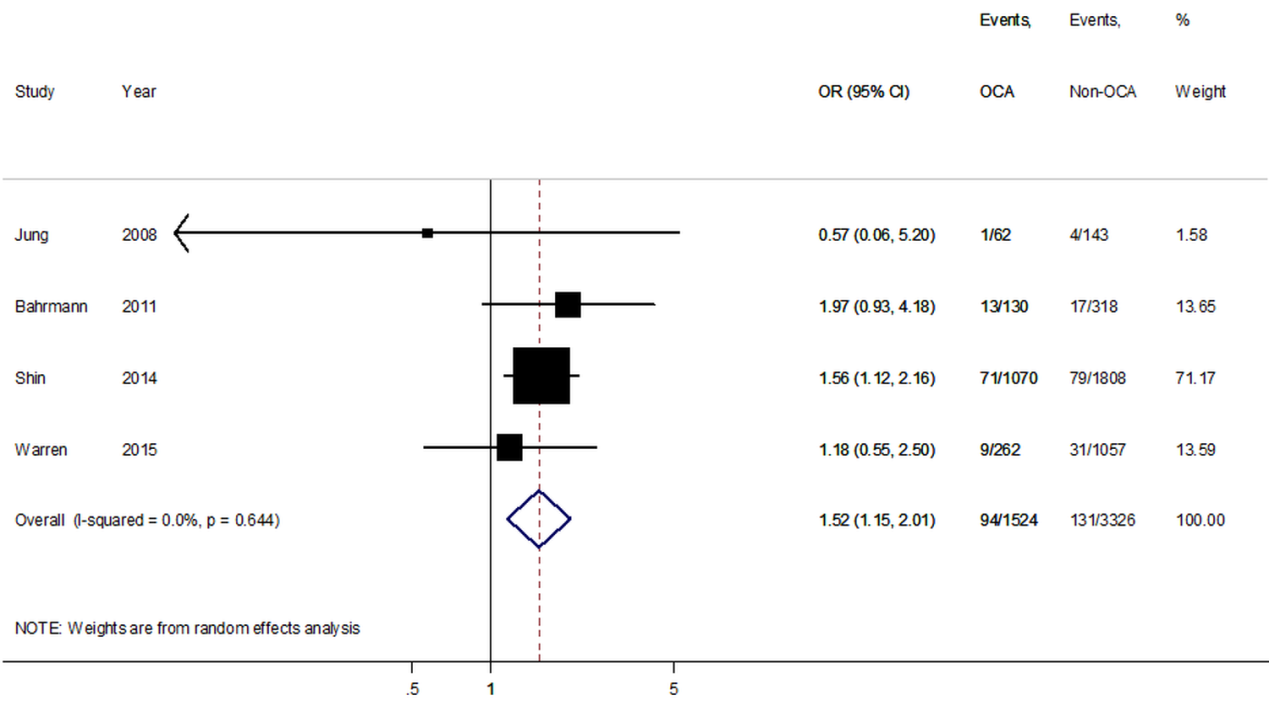


# Figure S17: The pooled odds ratio for recurrent myocardial infarction among patients with non-ST elevation myocardial infarction and occluded culprit artery compared with those with non-occluded culprit artery using the TIMI flow 0/1 as the definition of occluded culprit artery.


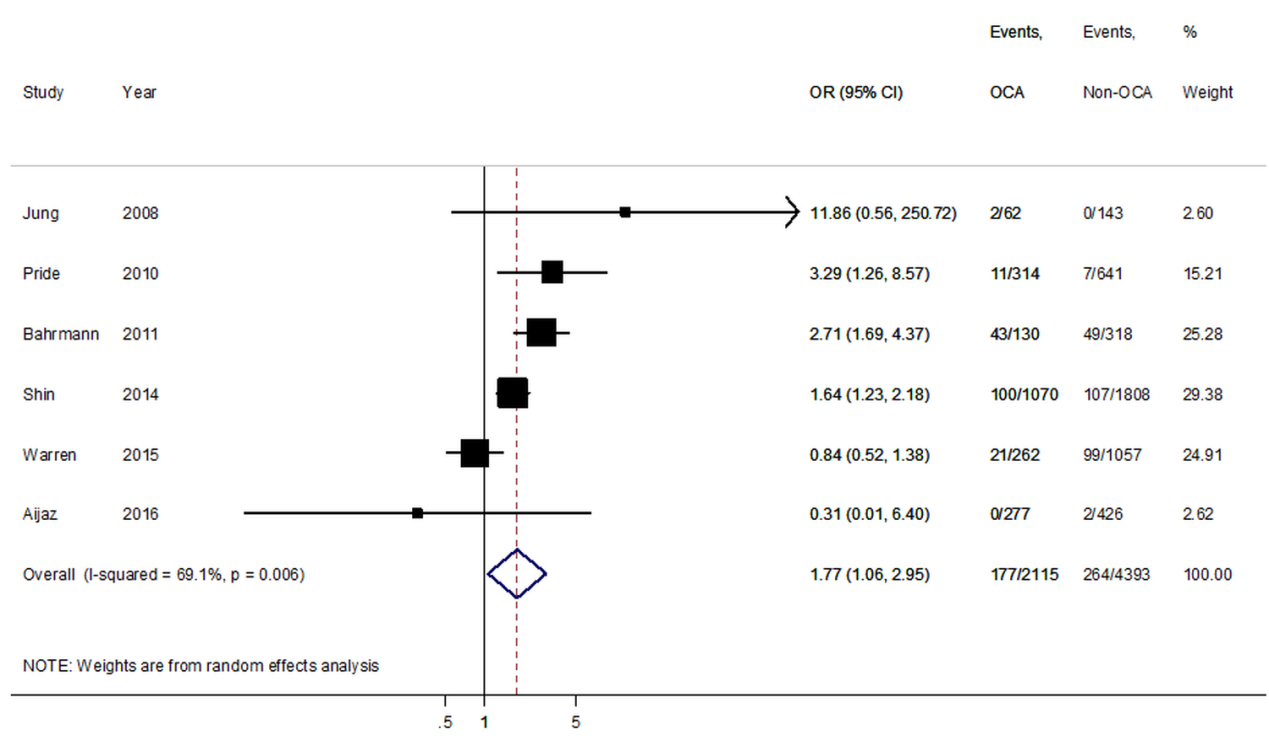


# Figure S18: The pooled odds ratio for recurrent myocardial infarction among patients with non-ST elevation myocardial infarction and occluded culprit artery compared with those with non-occluded culprit artery using the TIMI flow 0/1 as the definition of occluded culprit artery and time to coronary angiography within 28 days.

.
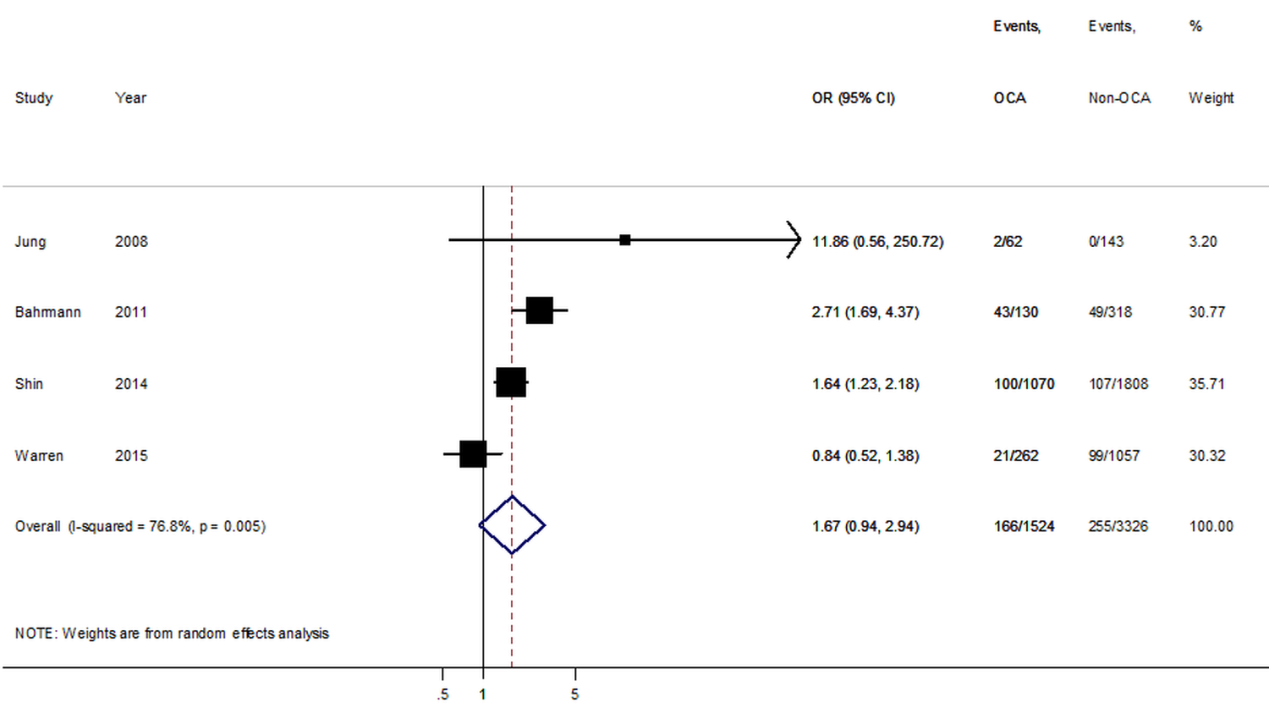


# Table S1: Data on revascularization

|  | N | Revasculari- zation | PCI | PCI success | CABG | Stent number | Stent length | DES |
| --- | --- | --- | --- | --- | --- | --- | --- | --- |
| Dixon et al [5] | | | | | | | | |
| OCA | 7199 | 7199  (100%) | 7199  (100%) | na | na | na | na | na |
| Non-OCA | 23187 | 23187  (100%) | 23187  (100%) | na | na | na | na | na |
| Bahrmann et al [11] | | | | | | | | |
| OCA | 130 | 116  (89.2%) | 100  (76.9%) | na | 16  (12.3%) | 1.5±  1.4 | 26.8±25.6 | 38  (29.2%) |
| Non-OCA | 318 | 247  (77.7%) | 213  (66.9%) | na | 34  (10.7%) | 1.0±  1.0 | 17.5±16.9 | 65  (20.4%) |
| Kim et al [7] | | | | | | | | |
| OCA | 665 | 665  (100%) | 665  (100%) | 611  (91.9%) | na | 1.58±0.89 | 25.1±6.4 | 521  (78.3%) |
| Non-OCA | 1429 | 1429  (100%) | 1429  (100%) | 1370  (95.9%) | na | 1.65±0.93 | 24.3±6.5 | 1217  (85.2%) |
| Soon et al [21] | | | | | | | | |
| OCA | 34 | 14  (41.1%) | 14  (41.1%) | 13  (38.2%) | 0 | na | na | na |
| Non-OCA | 109 | 55  (50.4%) | 55  (50.4%) | 54  (49.5%) | 0 | na | na | na |
| Shin et al [8] | | | | | | | | |
| OCA | 1070 | 1070  (100%) | 1070  (100%) | 1033  (96.5%) | na | 1.6±0.7 | 26.8±9.8 | 982  (91.8%) |
| Non-OCA | 1808 | 1808  (100%) | 1808  (100%) | 1756  (97.1%) | na | 1.6±0.9 | 25.4±10.7 | 1680  (92.9%) |
| Aijaz et al [22] | | | | | | | | |
| OCA | 277 | 125  (45.1%) | 60  (21.6%) | na | 66  (23.8%) | na | na | na |
| Non-OCA | 426 | 209  (49.1%) | 170  (39.9%) | na | 40  (9.4%) | na | na | na |

*CABG, coronary artery bypass graft; DES, drug-eluting stent; OCA, occlusive culprit artery; PCI, percutaneous coronary intervention
